# Developmental nicotine exposure engenders intergenerational downregulation and aberrant posttranslational modification of cardinal epigenetic factors in the frontal cortices, striata, and hippocampi of adolescent mice

**DOI:** 10.1186/s13072-020-00332-0

**Published:** 2020-03-05

**Authors:** Jordan M. Buck, Heidi C. O’Neill, Jerry A. Stitzel

**Affiliations:** 1 Institute for Behavioral Genetics, University of Colorado, 1480 30th Street, Boulder, CO 80309-0447 USA; 2 Department of Integrative Physiology, University of Colorado, Boulder, USA

**Keywords:** Nicotine, Neurodevelopment, Multigenerational, DNMT3A, TET2, MeCP2, HDAC2

## Abstract

**Background:**

Maternal smoking of traditional or electronic cigarettes during pregnancy, which constitutes developmental nicotine exposure (DNE), heightens the risk of neurodevelopmental disorders including ADHD, autism, and schizophrenia in children. Modeling the intergenerationally transmissible impacts of smoking during pregnancy, we previously demonstrated that both the first- and second-generation adolescent offspring of nicotine-exposed female mice exhibit enhanced nicotine preference, hyperactivity and risk-taking behaviors, aberrant rhythmicity of home cage activity, nicotinic acetylcholine receptor and dopamine transporter dysfunction, impaired furin-mediated proBDNF proteolysis, hypocorticosteronemia-related glucocorticoid receptor hypoactivity, and global DNA hypomethylation in the frontal cortices and striata. This ensemble of multigenerational DNE-induced behavioral, neuropharmacological, neurotrophic, neuroendocrine, and DNA methylomic anomalies recapitulates the pathosymptomatology of neurodevelopmental disorders such as ADHD, autism, and schizophrenia. Further probing the epigenetic bases of DNE-induced multigenerational phenotypic aberrations, the present study examined the expression and phosphorylation of key epigenetic factors via an array of immunoblot experiments.

**Results:**

Data indicate that DNE confers intergenerational deficits in corticostriatal DNA methyltransferase 3A (DNMT3A) expression accompanied by downregulation of methyl-CpG-binding protein 2 (MeCP2) and histone deacetylase 2 (HDAC2) in the frontal cortices and hippocampi, while the expression of ten-eleven translocase methylcytosine dioxygenase 2 (TET2) is unaltered. Moreover, DNE evokes multigenerational abnormalities in HDAC2 (Ser^394^) but not MeCP2 (Ser^421^) phosphorylation in the frontal cortices, striata, and hippocampi.

**Conclusions:**

In light of the extensive gene regulatory roles of DNMT3A, MeCP2, and HDAC2, the findings of this study that DNE elicits downregulation and aberrant posttranslational modification of these factors in both first- and second-generation DNE mice suggest that epigenetic perturbations may constitute a mechanistic hub for the intergenerational transmission of DNE-induced neurodevelopmental disorder-like phenotypes.

## Background

One in ten women in the United States disclose smoking conventional cigarettes during pregnancy, and 14% report consuming electronic cigarette during pregnancy [1, 2]. Concerningly, epidemiological studies reveal that, irrespective of the lack of corroborative research, the majority of individuals queried mistakenly believe that electronic cigarettes constitute a safer and healthier surrogate for conventional cigarettes, and this misperception is most frequent in women of reproductive age [3–7]. In actuality, the consumption of either conventional or electronic cigarettes during pregnancy constitutes developmental nicotine (NIC) exposure (DNE), which is linked to myriad fetal consequences including pre-mature birth, low birth weight, and sudden infant death syndrome [8–12]. In conjunction with its deleterious consequences for the newborn fetus, DNE also disrupts neurodevelopment and is associated with neurodevelopmental disorders such as ADHD, autism, and schizophrenia [1, 10–19]. Of particular concern, the neurodevelopmental consequences of DNE appear transmissible across multiple offspring generations, as demonstrated by a recent report indicating that grand-maternal smoking enhances the likelihood of autism diagnosis in grandchildren [17]. In support and expansion of previous epidemiological and animal model research, we recently reported that DNE precipitates multigenerational neurodevelopmental disorder-like hyperactivity, risk-taking behaviors and circadian disruptions as well as neurobiological perturbations in the frontal cortex and striatum including nicotinic acetylcholine receptor (nAChR) and DA transporter (DAT) dysfunction, proBDNF–BDNF imbalance, furin downregulation, and glucocorticoid receptor hypoactivity accompanied by hypocorticosteronemia [20–40]. Collectively, these findings provide clinical and preclinical evidence of the multigenerational predisposition to neurodevelopmental disorders conferred by maternal smoking during pregnancy and implicate DNE-induced alterations in corticostriatal acetylcholine, dopamine, BDNF, and glucocorticoid signaling therein.

DNA methylation is a fundamental epigenetic regulator of gene expression which modulates behavior, influences cholinergic, dopaminergic, neurotrophic, and glucocorticoid signaling, and is disrupted in neurodevelopmental disorders, DNE children, and animal models thereof [40–66]. For instance, DNA methylation levels at birth are inversely related to symptom severity in pediatric ADHD patients, and the children of maternal smokers exhibit global and locus-specific alterations in dorsolateral prefrontal cortical DNA methylation patterns that are directly linked to impaired neurodevelopment, deficient neurodifferentiation, atypical neuromorphology, and aberrant synaptogenesis [51, 53–58, 65, 66]. Similarly, DNA methylomic alterations are also linked to BDNF and HPA axis deficits in neurodevelopmental disorders as well as in DNE children and animal models [32–34, 36–38, 67–70]. Building upon this research, we recently documented corticostriatal global DNA methylome deficits in first- and second-generation adolescent DNE mice that co-occur with the multigenerational transmission of neurodevelopmental disorder-like behavioral perturbations, nAChR and DAT dysfunction, proBDNF/BDNF imbalance, furin deficits, and atypical glucocorticoid receptor activity [39, 40]. In light of this evidence, perturbed DNA methylation patterns represent a putative mechanistic basis for the predisposition to neurodevelopmental disorders in the children and grandchildren of maternal smokers.

Contemporary research indicates that DNE alters the transcription and expression of the epigenetic factors DNA methyltransferase 3A (DNMT3A) and ten-eleven translocase methylcytosine dioxygenase 2 (TET2) in rodent offspring [59, 71, 72]. Given that DNMT3A and TET2 reciprocally regulate DNA methylation via catalysis of de novo DNA methylation and demethylation, respectively, downregulation of DNMT3A and/or upregulation of TET2 could mediate the multigenerational global DNA methylome deficits and associated neurobehavioral phenotypes that we have previously identified in adolescent DNE mice [39, 40, 73, 74]. In support of this inference, alterations in corticostriatal and hippocampal DNMT3A and TET2 expression perturb the DNA methylome and thereby disrupt neurodevelopment, neuroplasticity, and synaptogenesis, elicit neurodegeneration, impair learning, memory, and cognition, and modify anxiety, stress responsivity, and emotional behaviors, and this cascade of DNMT3A and TET2 downregulation-induced DNA methylome perturbation occurs in neurodevelopmentally disordered and DNE children as well as animal models thereof [1, 35, 37, 38, 59, 67, 75–98]. Taken together, these findings raise the possibility that DNE may disrupt neurodevelopment in part by eliciting DNMT3A and/or TET2 dysregulation.

Beyond the presumptive roles of DNA-methylating and DNA-demethylating enzymes, the literature suggests that additional epigenetic factors may be involved in the intergenerational transmission of phenotypic aberrations elicited by developmental exposure to nicotine and other drugs of abuse [99, 100]. Therein, a recent study demonstrates that developmental morphine exposure elicits transgenerational alterations in the expression of methyl-CpG-binding protein-2 (MeCP2) and histone deacetylase 2 (HDAC2), the former of which (MeCP2) binds to methylated DNA and recruits the latter (HDAC2) to promote heterochromatization and epigenetic silencing of methylated loci [100–102]. Similar to DNMT3A and TET2 deregulation, aberrant corticostriatal and hippocampal MeCP2 and HDAC2 expression disrupts neurodevelopment, synaptogenesis, and synaptic plasticity, elicits BDNF and HPA axis dysfunction, impairs learning and memory, and alters stress responsivity, each of which phenomena are reported in neurodevelopmentally disordered and DNE children as well as animal models thereof [1, 36–38, 67, 81, 85, 86, 88–98, 102–117].

In addition to the putative roles of perturbed MeCP2 and HDAC2 expression in neurodevelopmental disorders, aberrant posttranslational modifications of MeCP2 and HDAC2 may alter the function of these epigenetic factors and thereby further contribute to neurodevelopmental disorders as well as the multigenerational impacts of DNE. Therein, MeCP2 (Ser^421^) phosphorylation facilitates neurodevelopment and neuronal function, experience-dependent chromatin remodeling, dendritic outgrowth and spine maturation, BDNF expression, and the neurocircuitry of behavior [118–120]. Moreover, HDAC2 (Ser^394^) phosphorylation disinhibits oxidative stress-induced neuroapoptosis and may thereby contribute to the aberrant neuroinflammatory and neurodegenerative processes implicated in the pathophysiology of ADHD, autism, and schizophrenia [106, 121–124]. In aggregate, the aforementioned evidence suggests putative roles for both the expression and posttranslational modification of MeCP2 and HDAC2 in the intergenerational neurodevelopmental disorder-like phenotypes elicited by DNE.

Collectively, prior studies implicate an ensemble of DNA methylomic and histonomic alterations in the etiology of ADHD, autism, and schizophrenia, in the heightened risk for neurodevelopmental disorders in DNE children and grandchildren, and in the ADHD-, autism-, and schizophrenia-like phenotypes exhibited by first-generation DNE offspring. While we have previously demonstrated corticostriatal global DNA hypomethylation in DNE offspring and grandoffspring, no prior studies have examined the multigenerational impacts of DNE on epigenetic factors such as DNA-methylating, DNA-demethylating, methylated DNA-binding, or histone-modifying proteins [39]. Given that neurodevelopmental disorders and DNE are associated with dysfunction of DNMT3A, TET2, MeCP2, and HDAC2, we posited that dysregulation of these proteins may contribute to the increased risk of neurodevelopmental disorders in DNE children and grandchildren as well as the neurodevelopmental disorder-like phenotypes exhibited by rodent DNE offspring and grandoffspring. Addressing this hypothesis, the current study characterized the multigenerational impacts of DNE on DNMT3A and TET2 expression as well as the expression and phosphorylation of MeCP2 and HDAC2 in the frontal cortices, striata, and hippocampi of first- and second-generation adolescent DNE mice.

## Results

**Fig. 1:**
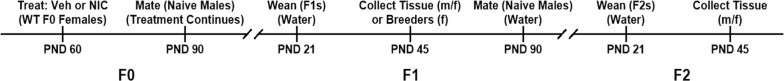
Procedural timeline for breeding and tissue collection. Beginning 30 days prior (PND 60) to crossing with drug-naïve sires (PND 90), C57BL/6J dams (zeroth generation, F0) underwent passive oral exposure to 0.2% saccharin (developmental vehicle exposure) or 0.2% saccharin containing 200 µg/mL nicotine (developmental nicotine exposure, DNE). Vehicle or nicotine treatment of F0 dams persisted through weaning of first-generation (F1) developmental vehicle-exposed (F1 Veh) or developmental nicotine-exposed (F1 NIC) offspring at PND 21. Thereafter, water was provided as the exclusive fluid source for all offspring. At PND 90, randomly selected female F1 NIC mice were crossed with drug-naïve sires to foster second-generation (F2) developmental nicotine-exposed (F2 NIC) offspring. To obtain tissue for subsequent immunoblot analyses, whole brains were extracted from PND 45 (adolescent) progeny belonging to each developmental exposure group (F1 Veh, F1 NIC, and F2 NIC), and bilateral frontal cortices, striata, and hippocampi were then collected by manual dissection. *PND* post-natal day, *Veh* 0.2% aqueous saccharin, *NIC* 200 µg/mL nicotine in 0.2% aqueous saccharin, *F1 Veh* first-generation developmental vehicle-exposed offspring, *F1 NIC* first-generation developmental nicotine-exposed offspring, *F2 NIC* second-generation developmental nicotine-exposed offspring

Previously described protocols for the breeding of mice and collection of tissues are diagrammed in Fig. 1 and detailed in “Methods” section [39, 40]. Briefly, F0 dams received either nicotine (200 µg/mL in 0.2% saccharin) or vehicle (0.2% saccharin) as the sole source of fluid beginning 30 days prior to mating with drug-naïve sires and continuing until the weaning of F1 pups. Analogous to smoking during pregnancy and nursing, first-generation DNE (F1 NIC) mice were thereby exposed to vehicle and nicotine from conception until weaning, while F1 Veh mice were exposed to vehicle alone on the same schedule. Second-generation DNE (F2 NIC) mice are the progeny of F1 NIC females bred with drug-naïve males. By this design, F2 NIC mice underwent exclusively indirect nicotine exposure via the maternal germline (oocytes). All experiments utilized tissue from both sexes obtained at PND 45, and sex was included as a biological variable for all data analyses. No main effect of sex was detected for any measure, and all datasets were therefore collapsed by sex.

### DNE confers multigenerational DNMT3A deficits in the frontal cortices and striata

**Fig. 2:**
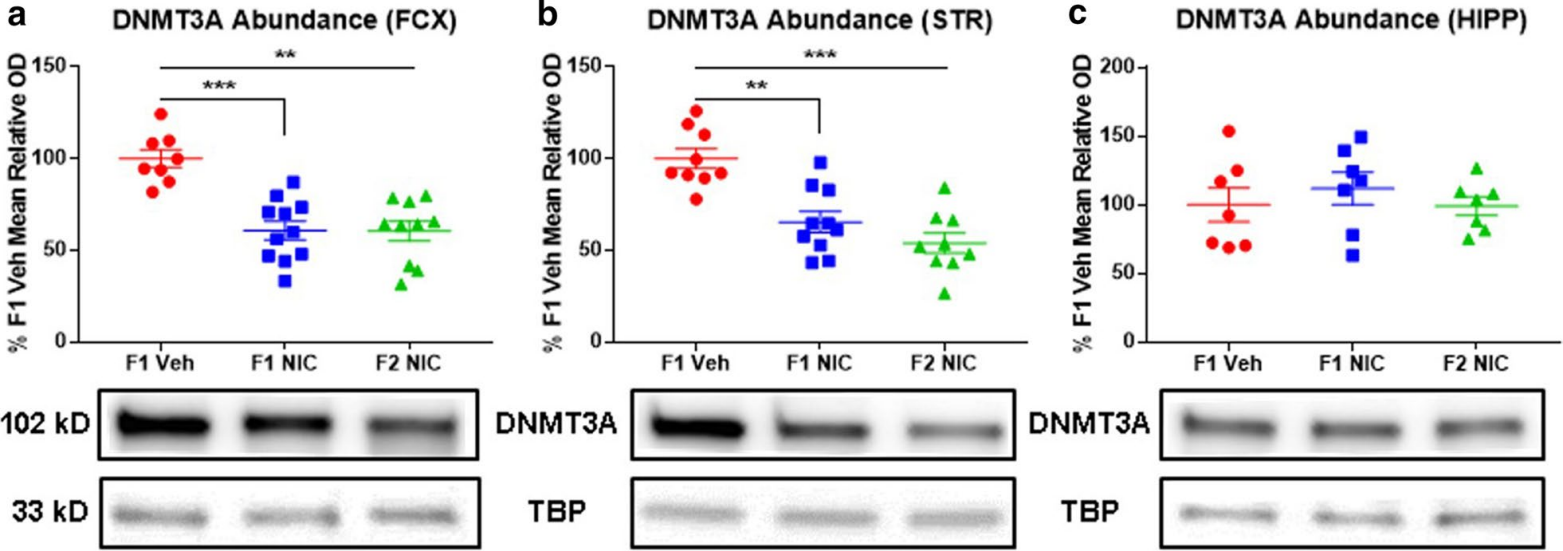
DNE elicits multigenerational DNMT3A deficits in the frontal cortices and striata. Representative Western blot images and densitometric measurements of DNMT3A abundance in frontal cortices (*n*_F1Veh_ = 8, *n*_F1NIC_ = 11, and *n*_F2NIC_ = 10), striata (*n*_F1Veh_ = 9, *n*_F1NIC_ = 10, and *n*_F2NIC_ = 9), and hippocampi (*n*_F1Veh_ = 7, *n*_F1NIC_ = 7, and *n*_F2NIC_ = 7). **a** DNMT3A abundance in frontal cortices. F1 NIC and F2 NIC mice have reduced frontal cortical DNMT3A content. **b** DNMT3A abundance in striata. F1 NIC and F2 mice NIC have reduced striatal DNMT3A content. **c** DNMT3A abundance in hippocampi. Hippocampal DNMT3A content is unaltered in F1 NIC and F2 NIC mice. *FCX* frontal cortices, *STR* striata, *HIPP* hippocampi, *DNMT3A* DNA methyltransferase 3A, *TBP* TATA-binding protein. All data are mean ± SEM. *p < 0.05; **p < 0.01; ***p < 0.001; ****p < 0.0001

DNMT3A maintains and establishes DNA methylation marks, and previous research reveals co-occurring DNMT3A deficits and global DNA hypomethylation in neurodevelopmental disorder-related brain regions of first-generation DNE mice [59, 73]. Therefore, we suspected that the global DNA methylome deficits which we previously reported in first- and second-generation DNE progeny may stem from the multigenerational transmission of DNE-induced DNMT3A deficits [39]. To test this hypothesis, we performed immunoblot analyses to compare DNMT3A abundance, expressed as percentage relative optical density versus the mean F1 Veh control value, in the frontal cortices, striata, and hippocampi of first- and second-generation adolescent DNE mice versus F1 Veh controls. Main effects of group (*F*_2,69_ = 13.1; *p* = 0.00001), region (*F*_2,69_ = 16.9; *p* = 0.000002), and a significant group × region interaction (*F*_4,69_ = 4.6; *p* = 0.003) were detected. Relative to F1 Veh mice, F1 NIC and F2 NIC mice exhibit DNMT3A downregulation in the (Fig. 2a) frontal cortices (*p* = 0.0009 and *p* = 0.001, respectively) and (Fig. 2b) striata (*p* = 0.008 and *p* = 0.0005, respectively), but no DNE-induced alterations in DNMT3A abundance were identified in the hippocampi (Fig. 2c).

### DNE does not impact TET2 content in the frontal cortices, striata, or hippocampi

**Fig. 3:**
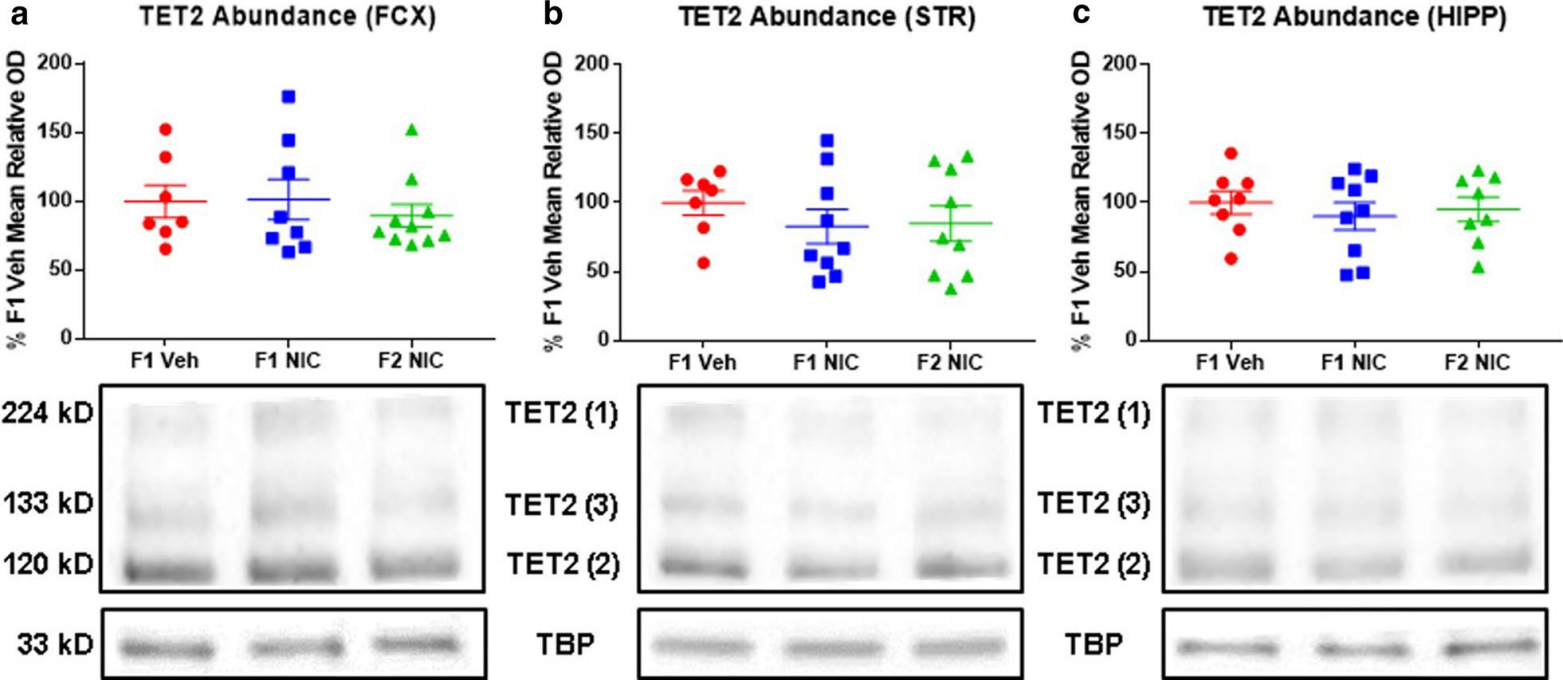
DNE does not impact TET2 content in the frontal cortices, striata, or hippocampi. Representative Western blot images and densitometric measurements of TET2 abundance in frontal cortices (*n*_F1Veh_ = 7, *n*_F1NIC_ = 8, and *n*_F2NIC_ = 10), striata (*n*_F1Veh_ = 7, *n*_F1NIC_ = 9, and *n*_F2NIC_ = 9), and hippocampi (*n*_F1Veh_ = 8, *n*_F1NIC_ = 9, and *n*_F2NIC_ = 8). **a** TET2 abundance in frontal cortices. Frontal cortical TET2 content is unaltered in F1 NIC and F2 NIC mice. **b** TET2 abundance in striata. Striatal TET2 content is unaltered in F1 NIC and F2 NIC mice. **c** TET2 abundance in hippocampi. Hippocampal TET2 content is unaltered in F1 NIC and F2 NIC mice. *FCX* frontal cortices, *STR* striata, *HIPP* hippocampi, *TET2* ten-eleven translocase methylcytosine dioxygenase 2, *TET2 (1)* TET2 isoform 1, *TET2 (2)* TET2 isoform 2, *TET2 (3)* TET2 isoform 3, *TBP* TATA-binding protein. All data are mean ± SEM

The DNA demethylase TET2 is dysregulated in first-generation DNE rodents, and TET2 dysfunction is broadly linked to myriad epigenetic and phenotypic alterations which mirror those observed in neurodevelopmental disorders as well as first- and second-generation DNE offspring [39, 40, 72, 77–79]. Accordingly, we reasoned that DNE may elicit multigenerational overexpression of TET2 which could contribute to the multigenerational global DNA hypomethylation which we previously reported in DNE mice [39]. Evaluating this prediction, we compared TET2 abundance in the frontal cortices (Fig. 3a), striata (Fig. 3b), and hippocampi (Fig. 3c) of adolescent DNE offspring and grandoffspring versus F1 Veh control mice. Contrary to our hypothesis, TET2 abundance in DNE mice did not differ from F1 Veh mice for any brain region assayed.

### DNE elicits multigenerational MeCP2 deficits in the frontal cortices and hippocampi

**Fig. 4:**
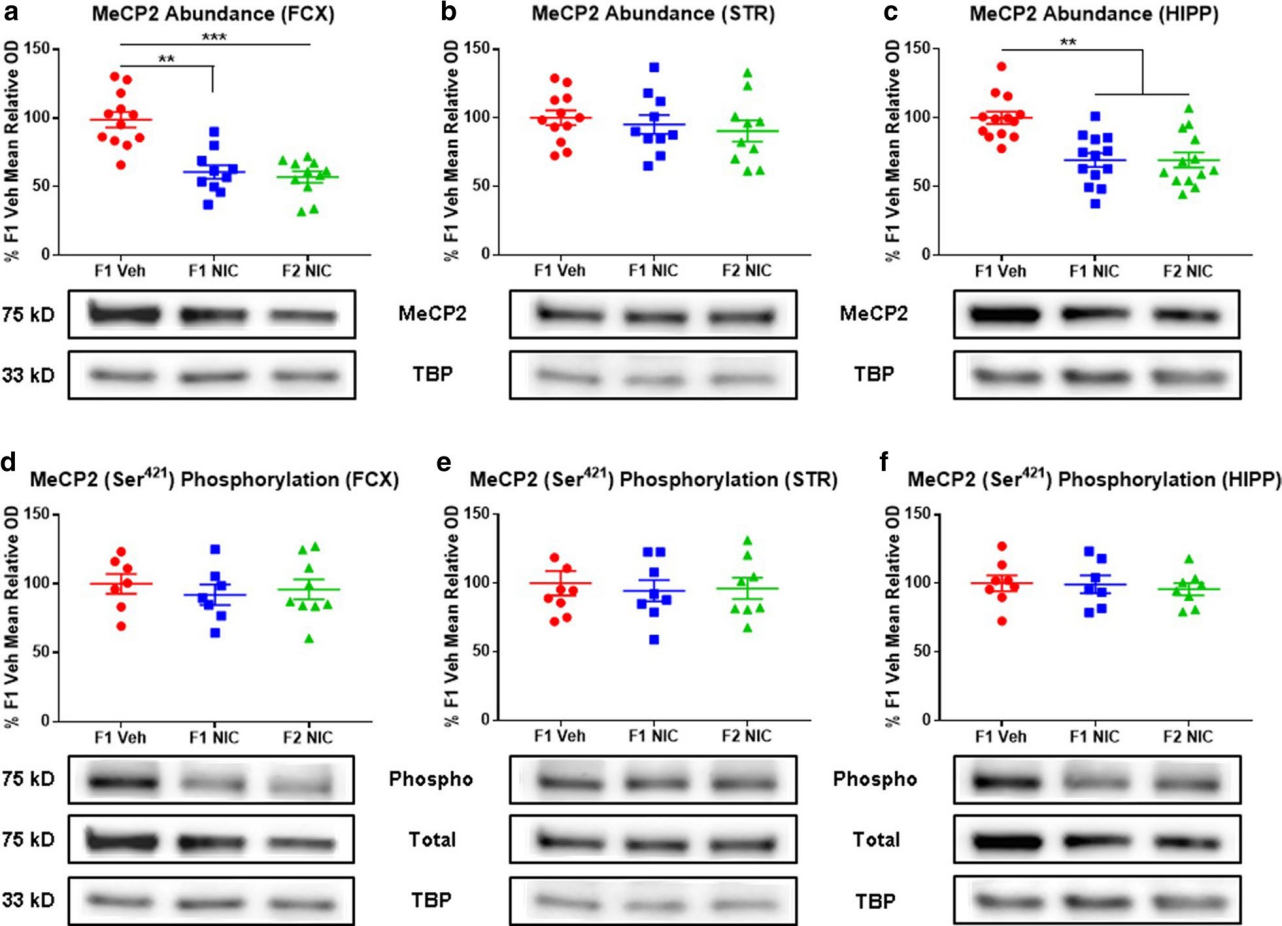
DNE elicits multigenerational MeCP2 deficits in the frontal cortices and hippocampi. Representative Western blot images and densitometric measurements of MeCP2 abundance and fractional MeCP2 (Ser^421^) phosphorylation in frontal cortices, striata, and hippocampi. **a** MeCP2 abundance in frontal cortices (*n*_F1Veh_ = 11, *n*_F1NIC_ = 10, and *n*_F2NIC_ = 11). F1 NIC and F2 NIC mice have reduced frontal cortical MeCP2 content. **b** MeCP2 abundance in striata (*n*_F1Veh_ = 12, *n*_F1NIC_ = 10, and *n*_F2NIC_ = 10). Striatal MeCP2 content is unaltered in F1 NIC and F2 NIC mice. **c** MeCP2 abundance in hippocampi (*n*_F1Veh_ = 13, *n*_F1NIC_ = 14, and *n*_F2NIC_ = 13). F1 NIC and F2 mice NIC have reduced hippocampal MeCP2 content. **d** Fractional MeCP2 (Ser^421^) phosphorylation in frontal cortices (*n*_F1Veh_ = 7, *n*_F1NIC_ = 6, and *n*_F2NIC_ = 7). Frontal cortical fractional MeCP2 (Ser^421^) phosphorylation is unaltered in F1 NIC and F2 NIC mice. **e** Fractional MeCP2 (Ser^421^) phosphorylation in striata (*n*_F1Veh_ = 8, *n*_F1NIC_ = 8, and *n*_F2NIC_ = 8). Striatal fractional MeCP2 (Ser^421^) phosphorylation is unaltered in F1 NIC and F2 NIC mice. **f** Fractional MeCP2 (Ser^421^) phosphorylation in hippocampi (*n*_F1Veh_ = 8, *n*_F1NIC_ = 6, and *n*_F2NIC_ = 9). Hippocampal fractional MeCP2 (Ser^421^) phosphorylation is unaltered in F1 NIC and F2 NIC mice. *FCX* frontal cortices, *STR* striata, *HIPP* hippocampi, *MeCP2* methyl-CpG binding protein 2, *total* total MeCP2, *phospho* phospho-MeCP2 (Ser^421^), *TBP* TATA-binding protein. All data are mean ± SEM. *p < 0.05; **p < 0.01; ***p < 0.001; ****p < 0.0001

MeCP2 dysregulation elicits a broad spectrum of epigenetic, neurobehavioral, neurofunctional, neurotrophic, and neuroendocrine anomalies that are consistent with observations in neurodevelopmental disorders and DNE children [61, 107–109, 115, 116]. While the impacts of DNE on MeCP2 are heretofore unexplored, developmental morphine exposure is known to elicit transgenerational upregulation of MeCP2 accompanied by various behavioral perturbations [100]. In consideration of these findings and our prior documentation of multigenerational global DNA methylome deficits in DNE mice, we posited that DNE may alter MeCP2 expression in first- and second-generation adolescent progeny [39]. Addressing this hypothesis, we assessed MeCP2 abundance in the frontal cortices, striata, and hippocampi of first- and second-generation adolescent DNE mice versus F1 Veh controls. Main effects of group (*F*_2,36_ = 16.2; *p* = 0.00001), region (*F*_2,59_ = 7.45; *p* = 0.045), and a significant group × region × measure interaction (*F*_2,59_ = 7.61; *p* = 0.030) were detected. Compared to F1 Veh mice, F1 NIC and F2 NIC mice have reduced MeCP2 content in the (Fig. 4a) frontal cortices (*p* = 0.003 and *p* = 0.0006, respectively) and (Fig. 4c) hippocampi (*p* = 0.004 and *p* = 0.005, respectively), but not in the striata (Fig. 4b).

### DNE does not impact MeCP2 (Ser^421^) phosphorylation in the frontal cortices, striata, or hippocampi

Of further relevance to neurodevelopmental disorders and the multigenerational impacts of DNE, MeCP2 (Ser^421^) phosphorylation regulates neurodevelopment and neuronal function, experience-dependent chromatin remodeling, dendritic outgrowth and spine maturation, BDNF expression, and the neural circuitry of behavior [118–120]. Accordingly, we inferred that DNE may elicit multigenerational perturbations in MeCP2 (Ser^421^) phosphorylation. Examining this possibility, we quantified fractional MeCP2 (Ser^421^) phosphorylation in the frontal cortices, striata, and hippocampi of DNE offspring and grandoffspring compared to F1 Veh control mice. Discordant with our hypothesis, no DNE-induced alterations in MeCP2 (Ser^421^) phosphorylation were detected in the frontal cortices (Fig. 4d), striata (Fig. 4e), or hippocampi (Fig. 4f).

### DNE precipitates multigenerational HDAC2 deficits in the frontal cortices and hippocampi

**Fig. 5:**
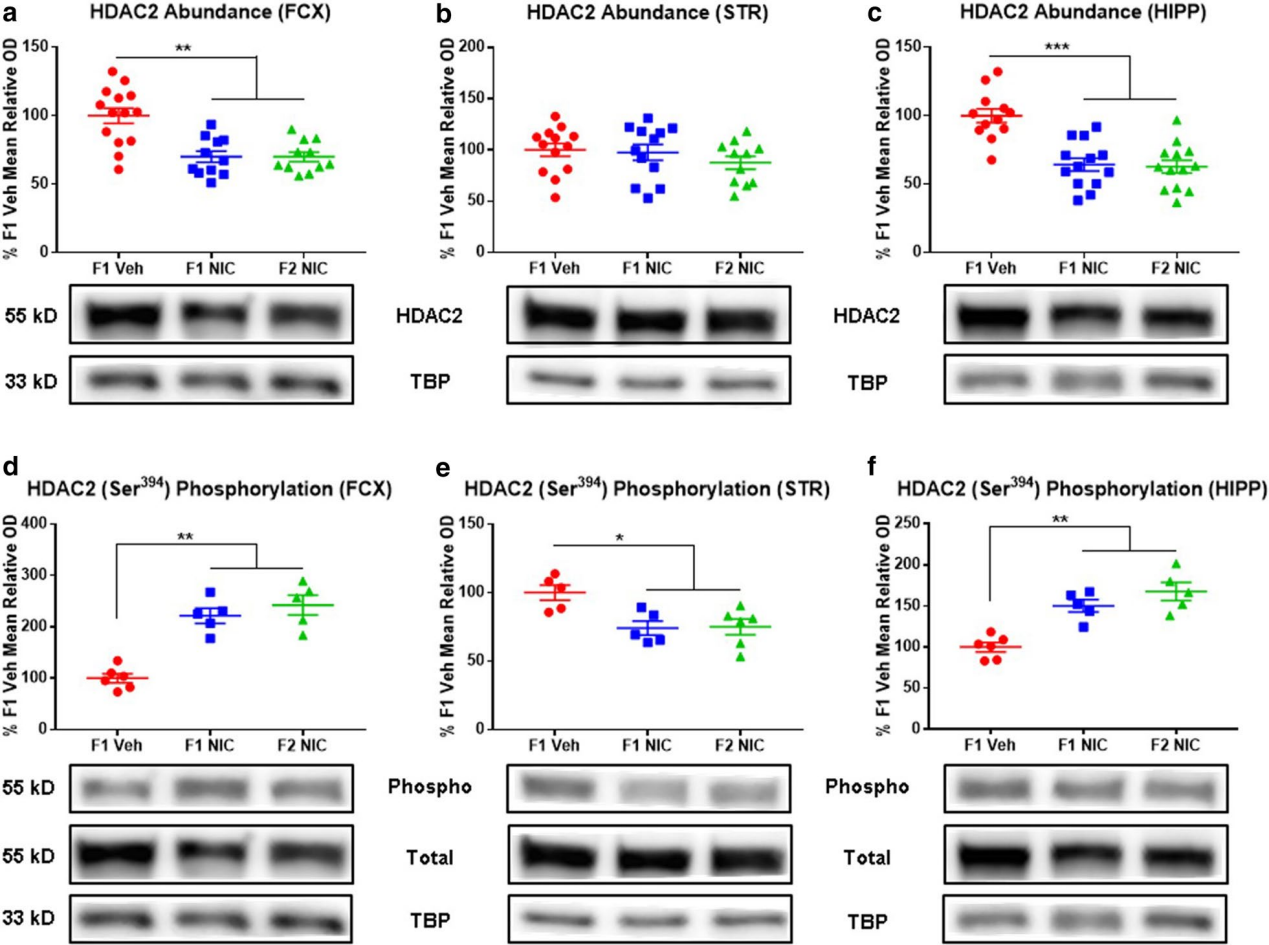
DNE elicits multigenerational HDAC2 deficits in the frontal cortices and hippocampi and alters HDAC2 (Ser^394^) phosphorylation in the frontal cortices, striata, and hippocampi. Representative Western blot images and densitometric measurements of HDAC2 abundance and fractional HDAC2 (Ser^394^) phosphorylation in frontal cortices, striata, and hippocampi. **a** HDAC2 abundance in frontal cortices (*n*_F1Veh_ = 14, *n*_F1NIC_ = 11, and *n*_F2NIC_ = 11). F1 NIC and F2 NIC mice have reduced frontal cortical HDAC2 content. **b** HDAC2 abundance in striata (*n*_F1Veh_ = 13, *n*_F1NIC_ = 12, and *n*_F2NIC_ = 12). Striatal HDAC2 content is unaltered in F1 NIC and F2 NIC mice. **c** HDAC2 abundance in hippocampi (*n*_F1Veh_ = 12, *n*_F1NIC_ = 13, and *n*_F2NIC_ = 13). F1 NIC and F2 NIC mice have reduced hippocampal HDAC2 content (**d**) Fractional HDAC2 (Ser^394^) phosphorylation in frontal cortices (*n*_F1Veh_ = 6, *n*_F1NIC_ = 5, and *n*_F2NIC_ = 5). F1 NIC and F2 NIC mice have increased frontal cortical fractional HDAC2 (Ser^394^) phosphorylation. **e** Fractional HDAC2 (Ser^394^) phosphorylation in striata (*n*_F1Veh_ = 5, *n*_F1NIC_ = 6, and *n*_F2NIC_ = 5). F1 NIC and F2 NIC mice have reduced striatal fractional HDAC2 (Ser^394^) phosphorylation. **f** Fractional HDAC2 (Ser^394^) phosphorylation in hippocampi (*n*_F1Veh_ = 6, *n*_F1NIC_ = 5, and *n*_F2NIC_ = 5). F1 NIC and F2 NIC mice have increased hippocampal fractional HDAC2 (Ser^394^) phosphorylation. *FCX* frontal cortices, *STR* striata, *HIPP* hippocampi, *HDAC2* histone deacetylase 2, *total* total HDAC2, *Phospho* phospho-HDAC2 (Ser^394^), *TBP* TATA-binding protein. All data are mean ± SEM. *p < 0.05; **p < 0.01; ***p < 0.001; ****p < 0.0001

Analogous to MeCP2 dysfunction, deregulation of HDAC2 precipitates an ensemble of neurobehavioral and neurodevelopmental perturbations that recapitulate key pathophysiological domains of multiple neurodevelopmental disorders and have been documented in DNE children and animal models [102, 104, 114]. While no studies have directly examined the impacts of DNE on HDAC2, developmental morphine exposure is known to elicit transgenerational downregulation of HDAC2 in conjunction with aberrant behavioral phenotypes [100]. Furthermore, HDAC2 participates in a DNA methylation–MeCP2–HDAC2 interactome. Taken together with these observations, our prior findings of DNE-induced multigenerational corticostriatal global DNA methylome deficits coupled with the elucidation of MeCP2 deficits in the present study led us to postulate that DNE may evoke multigenerational alterations in HDAC2 abundance [39]. Assessing this prediction, we compared HDAC2 abundance in the frontal cortices, striata, and hippocampi of first- and second-generation adolescent DNE mice versus F1 Veh controls. Main effects of group (*F*_2,37_ = 12.7; *p* = 0.00006), region (*F*_2,64_ = 12.47; *p* = 0.002), measure (*F*_1,64_ = 82.74; *p* < 0.0001), a significant group × region interaction (*F*_4,64_ = 4.93; *p* = 0.02), a significant group × measure interaction (*F*_2,64_ = 20.28; *p* = 0.001), a significant region × measure interaction (*F*_2,64_ = 40.23; *p* < 0.0001), and a significant group × region × measure interaction (*F*_4,64_ = 9.98; *p* = 0.002) were detected. Compared to F1 Veh mice, F1 NIC and F2 NIC mice display decreased HDAC2 abundance in the (Fig. 5a) frontal cortices (*p* = 0.007 and *p* = 0.005, respectively) and (Fig. 5c) hippocampi (*p* = 0.009 and *p* = 0.0006, respectively), while no DNE-induced alterations in HDAC2 content were apparent in the striata (Fig. 5b).

### DNE manifests multigenerational alterations in HDAC2 (Ser^394^) phosphorylation in the frontal cortices, striata, and hippocampi

Of additional pertinence to neurodevelopmental disorders and the multigenerational impacts of DNE, phospho-HDAC2 (Ser^394^) derepresses oxidative neuroinflammation and neuroapoptosis and may thereby contribute to the pathophysiology of ADHD, autism, and schizophrenia [106, 121–124]. Therefore, we next assessed the hypothesis that DNE elicits multigenerational perturbations in HDAC2 (Ser^394^) phosphorylation. To this end, immunoblots were conducted to assess fractional HDAC2 (Ser^394^) phosphorylation in the frontal cortices, striata, and hippocampi of DNE offspring and grandoffspring compared to F1 Veh controls. In contrast to F1 Veh mice, F1 NIC and F2 NIC mice have increased fractional HDAC2 (Ser^394^) phosphorylation in the (Fig. 5d) frontal cortices (*p* = 0.002 and *p* = 0.006, respectively) and (Fig. 5f) hippocampi (*p* = 0.007 and *p* = 0.009, respectively) as well as reduced fractional HDAC2 (Ser^394^) phosphorylation in the (Fig. 5e) striata (*p* = 0.045 and *p* = 0.047, respectively).

## Discussion

**Fig. 6:**
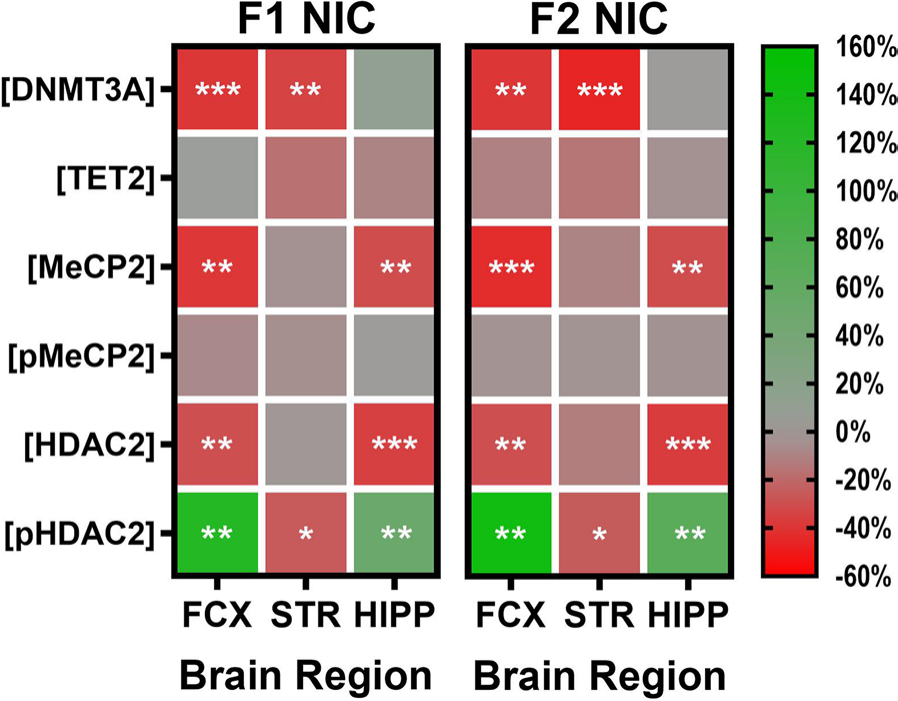
Compendium of results. Dual-gradient heatmaps depicting the percentage differences relative to F1 Veh control mice in each outcome measure (vertical axis) and brain region (horizontal axis) for F1 NIC (left) and F2 NIC (right) mice. A percentage difference of zero indicates no difference (depicted in gray) relative to F1 Veh control mice, whereas percentage differences of − 60% and 160% indicate a 60% decrease (depicted in red) and a 160% increase (depicted in green), respectively, relative to F1 Veh control mice. *F1 NIC* first-generation developmental nicotine-exposed adolescent offspring, *F2 NIC* second-generation developmental nicotine-exposed adolescent offspring, *FCX* frontal cortices, *STR* striata, *HIPP* hippocampi, *DNMT3A* DNA methyltransferase 3A, *TET2* ten-eleven translocase methylcytosine dioxygenase 2, *MeCP2* methyl-CpG-binding protein 2, *pMeCP2* phospho-MeCP2 (Ser^421^), *HDAC2* histone deacetylase 2, *pHDAC2* phospho-HDAC2 (Ser^394^). *p < 0.05; **p < 0.01; ***p < 0.001

Toward an improved understanding of the epigenetic mechanisms underlying the association of DNE with neurodevelopmental disorders in children and grandchildren, the present study addresses a void in the literature concerning the hitherto unknown multigenerational impacts of DNE on DNMT3A, TET2, MeCP2, and HDAC2 expression as well as MeCP2 (Ser^421^) and HDAC2 (Ser^394^) phosphorylation in the adolescent frontal cortices, striata, and hippocampi. Importantly, a noteworthy limitation in the experimental design of this study is the omission of a second-generation developmental vehicle-exposed control group for direct comparison to second-generation DNE mice. However, prior independent research detected no intergenerational impacts of developmental saccharin (vehicle) exposure on various neurodevelopmental disorder-related phenotypes such as locomotor activity, risk-taking behaviors, and dopamine signaling, the latter of which findings are corroborated by preliminary data from our lab [125]. A synopsis of the findings of this study is portrayed in Fig. 6.

Neurodevelopmentally disordered as well as DNE children and rodents exhibit analogous DNA methylome alterations which appear to contribute to the neurobehavioral, neurotrophic, and neuroendocrine disruption characteristic of neurodevelopmental disorders, the children of maternal smokers, and animal models thereof [32–39, 46–59]. Moreover, previous research suggests that dysfunction of the DNA methyltransferase DNMT3A may underlie DNA methylome aberrations such as those which we previously reported in the frontal cortices and striata of first- and second-generation adolescent DNE mice [39, 59, 71]. Addressing this possibility, the current study is the first to demonstrate DNE-induced downregulation of DNMT3A in the frontal cortices and striata, but not the hippocampi, of first- and second-generation adolescent progeny. These findings suggest that DNE exerts regioselective impacts on DNMT3A expression that may stem from the differential distribution and/or subunit composition of nAChRs among the brain regions assessed herein, which could in turn confer increased nicotine sensitivity and/or responsivity of DNMT3A expression in the frontal cortices and striata relative to the hippocampi. The brain region-selective multigenerational effects of DNE on DNMT3A expression could thereby conduce a discrete subset of multigenerational DNE-induced phenotypes which are more attributable to corticostriatal versus hippocampal dysfunction, such as our previous findings of hyperactivity and risk-taking behaviors as well as corticostriatal but not hippocampal proBDNF/BDNF imbalance and global DNA hypomethylation [39, 40]. However, future research is necessary to comprehensively map the brain regional selectivity of the multigenerational impacts of DNE on DNMT3A expression and to determine the specific behavioral and neurobiological consequences thereof.

From a translational perspective, the results of this study suggest that DNE-induced downregulation of corticostriatal DNMT3A expression identified herein may contribute to the DNA methylome perturbations documented in neurodevelopmentally disordered and DNE children, but future clinical investigations are necessary to examine this potentiality [46–57]. These findings also warrant further research to assess the implicit possibility that DNE-elicited DNMT3A downregulation and associated epigenetic, neurotrophic, and HPA axis changes may be intergenerationally transmissible in humans as appears to be the case in mice.

Congruent with the putative role of DNMT3A downregulation in neurodevelopmental disorder-associated and DNE-evoked DNA methylome deficits, contemporary rodent research demonstrates deregulation of TET2, an enzyme which catalyzes DNA demethylation, accompanied by global DNA hypomethylation in peripheral tissues of first-generation DNE offspring [72]. However, the present study indicates that DNE does not impact frontal cortical, striatal, or hippocampal TET2 expression in either first- or second-generation DNE progeny. Taken together with the aforementioned DNE-induced corticostriatal DNMT3A deficits elucidated herein, the lack of detectable alterations in TET2 abundance in DNE offspring and grandoffspring results suggests that corticostriatal DNMT3A deficits principally underlie the multigenerational DNE-induced corticostriatal DNA methylome deficits and co-occurring phenotypes which we previously documented [39].

Similar to the consequences of DNMT3A and TET2 dysregulation as well DNA methylome anomalies, altered corticostriatal and hippocampal expression of the methylated DNA-binding protein MeCP2 confers neurodevelopmental and synaptoplastic as well as neurotrophic and HPA axis aberrations which mirror observations in neurodevelopmentally disordered and DNE children [1, 35–38, 67, 86, 88–98, 102–105, 107, 109]. While no prior studies have assessed the multigenerational impacts of DNE on MeCP2, the literature implicates MeCP2 deregulation in the intergenerationally transmissible phenotypes elicited by developmental exposure to morphine and other drugs of abuse [99, 100]. In furtherance of this line of research, the present study is the first to elucidate multigenerational downregulation of MeCP2 in the frontal cortices and hippocampi, but not the striata, of adolescent DNE mice. Interestingly, the downregulation of MeCP2 identified in DNE offspring and grandoffspring contrasts with the MeCP2 upregulation elicited by developmental morphine exposure, implying a degree of drug-selectivity in the direction of developmental drug exposure-induced changes in MeCP2 expression [100]. Moreover, while both DNMT3A and MeCP2 were downregulated in the frontal cortices, DNMT3A alone was downregulated in the striata, whereas MeCP2 alone was downregulated in the hippocampi of DNE offspring and grandoffspring. Taken together, these findings suggest that the intergenerational impacts of DNE on specific regulators of the DNA methylome are brain region-dependent and, by extension, may selectively contribute to a subset of DNE-evoked neurodevelopmental disorder-like phenotypes. Therein, the brain regional selectivity of the multigenerational impacts of DNE may arise from differential distribution and/or subunit composition of nAChRs among the unique cellular ensembles comprising each brain region assessed, but further research is necessary to elucidate the molecular biological bases as well as the phenotypic consequences of the brain regional selectivity of the multigenerational impacts of DNE on various epigenetic factors. Ultimately, these novel findings implicate DNE-induced MeCP2 downregulation in the ensemble of behavioral, neuropharmacological, neurotrophic, and HPA axis anomalies which we previously documented in first- and second-generation DNE mice. By extension, these results implicate MeCP2 deficits in the etiology of DNE-related neurodevelopmental disorders including ADHD, autism, and schizophrenia [39, 40].

Complementing the relevance of altered MeCP2 expression to neurodevelopmental disorders and the multigenerational consequences of DNE, atypical MeCP2 (Ser^421^) phosphorylation hinders neurodevelopment, experience-dependent chromatin remodeling, dendritic outgrowth and spine maturation, and BDNF expression, resulting in anomalous behavioral phenotypes which are concordant with ADHD, autism, schizophrenia, and smoking during pregnancy [118–120]. Despite the shared association of neurodevelopmental disorders and DNE with the neurobehavioral consequences of altered phospho-MeCP2 (Ser^421^) content, MeCP2 (Ser^421^) phosphorylation was unaltered in all brain regions of DNE mice assayed. These data indicate that aberrant phosphorylation of MeCP2 (Ser^421^) is not a mechanism whereby DNE elicits neurodevelopmental disorder-like brain and behavioral alterations.

Analogous to the perturbations elicited by dysregulation of DNMT3A, TET2, MeCP2, and the DNA methylome, aberrant HDAC2 expression in the brain impairs neurodevelopment, neurotrophic signaling, and HPA axis function in a manner consistent with observations in ADHD, autism, schizophrenia, and the children of maternal smokers [35–37, 67, 86, 88–98, 106, 110, 111, 126]. Similar to MeCP2, HDAC2 dysregulation is also implicated in the intergenerational phenotypic transmission precipitated by developmental exposure to morphine and other drugs of abuse [99, 100]. Expanding this area of research to capture the multigenerational impacts of DNE, this study is the first to identify HDAC2 deficits in the frontal cortices and hippocampi, but not the striata, of adolescent DNE offspring and grandoffspring. Notably, the downregulation of HDAC2 in the frontal cortices and hippocampi of DNE offspring and grandoffspring mirrors the brain regional selectivity of DNE-induced intergenerational MeCP2 downregulation as well as the opposite direction of change compared to the transgenerational HDAC2 upregulation previously reported in developmental morphine-exposed mice [100]. Possible explanations for the regioselectivity of intergenerational DNE-evoked HDAC2 downregulation again include variable nAChR expression patterns and/or subunit compositions across the brain regions assessed, which could differentially contribute to the neurodevelopmental disorder-like neurobehavioral anomalies exhibited by first- and second-generation DNE mice [39, 40]. Ultimately, these data are the first to implicate HDAC2 deficiency in the multigenerational transmission of DNE-evoked, behavioral, neuropharmacological, neurotrophic, and neuroendocrine perturbations which we have previously reported [39, 40].

Lastly, the current study is the first to demonstrate that phospho-HDAC2 (Ser^394^) content is elevated in the frontal cortices and hippocampi but decreased in the striata of both first- and second-generation adolescent DNE progeny. These results recapitulate the pathophysiology of ADHD, autism, and schizophrenia. Considering that HDAC2 (Ser^394^) hyperphosphorylation disinhibits oxidative stress-induced neuronal inflammation and apoptosis [106], these results imply that multigenerational DNE-induced alterations in HDAC2 (Ser^394^) phosphorylation may disinhibit neuroinflammation and oxidative neurodegeneration and thereby impede neurodevelopment in the frontal cortices and hippocampi, but not the striata [121–124].

## Conclusions

The results of the present study reveal that DNE precipitates intergenerational transmission of corticostriatal DNMT3A deficiency, downregulation of MeCP2 and HDAC2 in the frontal cortices and hippocampi, and aberrant corticostriatal and hippocampal HDAC2 (Ser^394^) phosphorylation, while TET2 expression and MeCP2 (Ser^421^) phosphorylation were unaltered. Cumulatively, these findings imply that DNE differentially impacts discrete epigenetic factors in a brain region-selective fashion. Moreover, the findings of this study suggest that DNE-induced, brain region-selective epigenetic alterations may comprise nexuses for the behavioral, neuropharmacological, neurotrophic, and neuroendocrine anomalies that we previously reported in DNE offspring and grandoffspring [39, 40]. From a translational frame of reference, the results of this study contribute to a burgeoning literature indicating that DNE confers an ensemble of neurodevelopmental disorder-like phenotypes that are intergenerationally transmitted via a putative epigenetic mechanism. However, as DNE-induced epigenetic perturbations exhibited both shared and distinct patterns across the brain regions assessed herein, future research is warranted to delineate the gene-, cell type-, and brain regional specificity of DNE-evoked multigenerational epigenetic alterations and the relevance to neurodevelopmental disorders thereof.

## Methods

### Animals

All experimental and housing conditions were reviewed and pre-authorized by the Institutional Animal Care and Utilization Committee at the University of Colorado Boulder and conform to the guidelines for animal care and use established by the NIH and the Guide for the Care and Use of Laboratory Animals (8th Ed.). All mice were maintained in the same animal facility on a standard 12 h light/dark cycle (lights on at 07:00) and were provided food (Envigo Teklad 2914 irradiated rodent diet, Harlan, Madison, WI) and water ad libitum. As diagrammed in Fig. 1 and previously described, beginning 30 days prior to mating with drug-naïve sires, C57BL/6J dams received 0.2% saccharin (ThermoFisher, Waltham, MA) (vehicle) or 0.2% saccharin and nicotine (MilliporeSigma, Burlington, MA) (200 µg/mL freebase) (DNE) in place of drinking water [39, 40]. Vehicle and nicotine solutions were replaced twice weekly, and treatment of dams continued until weaning of offspring at PND 21, whereafter water was provided to all progeny as the sole fluid source. Randomly selected female F1 DNE offspring were subsequently mated with drug-naïve sires to foster F2 DNE (maternal germline nicotine-exposed) offspring. Female F1 DNE mice used to breed F2 DNE progeny were naïve to direct (post-weaning) nicotine exposure; as such, DNE-elicited phenotypes are transmitted from F1 to F2 generation DNE offspring exclusively via the F1 maternal germline. All experiments utilized tissue collected at PND 45 (adolescence) from both sexes of offspring, and sex was included as a biological variable in statistical analyses. To minimize between-litter and between-breeder variability within each group, tissue obtained from a minimum of 6 total litters from a minimum of 4 total breeder pairs was assayed for each group and experiment. There were no group differences in litter size or pup survival rates, and no covariation with litter or breeder was detected for any dataset.

Importantly, the 200 µg/mL oral nicotine dosage utilized herein yields a nicotine pharmacokinetic profile in C57BL/6J mice which is comparable to that of regular smokers, confers behavioral and neurobiological anomalies, hinders neurodevelopment, and is widely implemented across the DNE literature, thereby augmenting the generalizability of the DNE paradigm employed herein [20, 30, 39, 40, 127–130]. It should also be noted that, upon co-housing with pre-treated female breeders, drug-naïve males gained access to vehicle or nicotine drinking solutions. Therefore, the DNE paradigm utilized herein may be more accurately classified as parental rather than exclusively maternal DNE for first-generation DNE offspring, whereas second-generation DNE mice were exposed to nicotine solely via the maternal germline (oocytes). Notably, first-generation developmental vehicle-exposed mice were used as controls for comparison to both first- and second-generation DNE mice, as pilot data and previous research reveal no intergenerational impacts of developmental saccharin exposure [125].

### Tissue collection

Tissue collection was performed as previously described [39, 40]. Briefly, intact brains were obtained after cervical dislocation and decapitation at PND 45, and bilateral frontal cortices, striata, and hippocampi were manually dissected in an ice-chilled glass dish.

### Tissue lysis and fractionation of nuclear-enriched proteins

Immediately following collection, tissue dissectants were homogenized and nuclear-enriched proteins fractionated using a NE-PER kit (ThermoFisher, Waltham, MA) according to manufacturer protocol.

### Determination and standardization of lysate protein concentrations

Protein concentrations for all nuclear-enriched lysates were determined using a Pierce bicinchoninic acid (BCA) Assay Kit (ThermoFisher, Waltham, MA) according to manufacturer protocol. Upon determination of protein concentration, samples were diluted to 2 µg/µL, separated into 15 µL (30 µg) aliquots, and stored at − 80 °C until subsequent immunoblot assays.

### Immunoblotting procedures

Immunoblot experiments were conducted via a standard protocol as previously described [40, 131, 132]. Briefly, lysate aliquots containing 30 µg total protein were reduced/denatured, loaded onto 4–20% polyacrylamide gradient tris–glycine gels (Bio-Rad, Hercules, CA), electrophoresed, and electroblot-transferred to 0.45 µm Immobilon-P PVDF membrane (MilliporeSigma, Burlington, MA). Following transfer, membranes were incubated at room temperature (RT) for 30 min in blocking solution containing 5% non-fat milk (Bio-Rad, Hercules, CA) (for total protein detection) or 5% bovine serum albumin (MilliporeSigma, Burlington, MA) (for phosphoprotein detection) with 3% normal donkey serum (MilliporeSigma, Burlington, MA) in Tris-buffered saline (pH 7.4) (ThermoFisher, Waltham, MA) with 0.15% Tween-20 (Bio-Rad, Hercules, CA) (0.15% TBST) to deter non-specific antibody binding. Upon completion of blocking, membranes were incubated overnight at 4 °C with agitation in primary antibody solution containing the appropriate primary antibody (raised in rabbit) diluted to a concentration of 1:1000 in blocking solution. Primary antibodies utilized for this study were as follows: anti-TBP (ProteinTech Cat# 22006-1-AP), anti-DNMT3A (ProteinTech Cat#10954-1-AP), anti-TET2 (ProteinTech Cat# 21207-1-AP), anti-MeCP2 (ProteinTech Cat# 10861-1-AP), anti-phospho-Ser^421^ MeCP2 (PhosphoSolutions Cat# p1205-421), anti-HDAC2 (ProteinTech Cat# 12922-1-AP), and anti-phospho-Ser^394^ HDAC2 (PhosphoSolutions Cat# p11421-394). After primary antibody incubation, membranes were washed 5 × 5 min in 0.15% TBST (with agitation). Following washing, membranes were incubated for one hour at RT (with agitation) in secondary antibody solution containing horseradish peroxidase-conjugated donkey anti-rabbit IgG (Bio-Rad, Hercules, CA) diluted to a concentration of 1:3000 in blocking solution. Following secondary antibody incubation, membranes were washed 5 × 5 min in 0.15% TBST with agitation, and were then incubated (without agitation) for 5 min in chemiluminescent substrate (Clarity Western ECL Substrate, Bio-Rad, Hercules, CA). The chemiluminescent signals emitted from all membranes were captured using a FluorChem Imager (ProteinSimple, San Jose, CA).

### Immunoblot densitometry

Unprocessed 8-bit immunoblot images were uploaded to ImageJ 1.52a for densitometry [133]. Background was subtracted from all images prior to measurement of mean grey values for target and loading control bands [40, 134]. Mean grey values obtained for target bands were divided by those for corresponding loading control bands to determine relative optical density of each target protein [40, 134]. To calculate fractional phosphorylation of MeCP2 (Ser^421^) and HDAC2 (Ser^394^), corresponding relative optical density values for the phospho-specific bands were divided by those for the pan-specific (total) bands. Notably, and consistent with previous reports, the anti-TET2 antibody used for this study detected three discrete TET2 isoforms [135]. However, no isoform-specific differences in TET2 abundance were detected, and thus the relative optical density values for each TET2 isoform (isoforms 1, 2, and 3) were summed to provide a measure of total TET2 relative optical density. To obtain biologically descriptive outcome measures, arbitrary relative optical density values for DNMT3A, total TET2, total MeCP2, and total HDAC2, as well as the fractional phosphorylation values for phospho-MeCP2 (Ser^421^) and phospho-HDAC2 (Ser^394^), were transformed to, respectively, percentages relative optical density and percentages fractional phosphorylation versus the mean control (F1 Veh) value for each sample [40, 134], in which form data were both analyzed and visualized.

### Statistical analyses

For DNMT3A and TET2 immunoblots, percentages relative optical density versus the mean F1 Veh control value were analyzed by mixed ANOVA with the between-subjects factor group (F1 Veh, F1 NIC, or F2 NIC) and the within-subjects factor region (frontal cortices, striata, or hippocampi). For total and phosphorylated MeCP2 and HDAC2 datasets, percentages relative optical density and percentages fractional phosphorylation versus the mean control (F1 Veh) value were analyzed by mixed ANOVA with the between-subjects factor group (F1 Veh, F1 NIC, or F2 NIC) and the within-subjects factors region (frontal cortices, striata, or hippocampi) and measure (total or phospho-MeCP2 for MeCP2 immunoblots; total or phospho-HDAC2 for HDAC2 immunoblots).

Statistical analyses were performed using SPSS (IBM Analytics, Armonk, NY), and data were visualized via GraphPad Prism 7.04 (GraphPad Software, La Jolla, California, USA). Prior to statistical analyses, data were screened for outliers using the ROUT test (*Q* = 1%), and verified outliers were omitted from analyses as indicated. A maximum of one outlier was excluded per group for each dataset. All data were first analyzed by multivariate ANOVA to assess potential effects of sex, breeder, or litter. No main effects of or interactions with these variables were detected for any outcome measure, and data were therefore collapsed accordingly.

## Data Availability

All data reported herein are available from the corresponding author upon reasonable request.
